# Transcriptomic response in *Acropora muricata* under acute temperature stress follows preconditioned seasonal temperature fluctuations

**DOI:** 10.1186/s13104-018-3230-z

**Published:** 2018-02-09

**Authors:** Sonny T. M. Lee, Shashank Keshavmurthy, Silvia Fontana, Mezaki Takuma, Wen-Hua Chou, Chaolun Allen Chen

**Affiliations:** 1 Section of Gastroenterology, Hepatology and Nutrition, Department of Medicine, University of Chicago Medicine, Chicago, IL USA; 2 Biodiversity Research Center, Academia Sinica, Taipei, Taiwan; 3 Taiwan International Graduate Program (TIGP)-Biodiversity, Academia Sinica, Taipei, Taiwan; 4 Institute of Oceanography, National Taiwan University, Taipei, Taiwan; 5 National Taiwan Normal University, Taipei, Taiwan; 6 Biological Institute on Kuroshio, Otsuki, Kochi Japan

**Keywords:** Coral, Temperature stress, Adaptation, Transcriptomics, hsp70, Phosphoenolpyruvate carboxykinase

## Abstract

**Objective:**

Global climate change has resulted in the decline of health and condition of various coral reefs worldwide. Here, we describe expression profiles of *Acropora muricata* collected during opposing seasons in Otsuki, Kochi, Japan to define the capacity of corals to cope with changing environmental conditions. Coral communities in Otsuki experience large temperature fluctuations between the winter (~ 16 °C) and summer (~ 27 °C).

**Results:**

Coral nubbins that were collected in the summer showed no change in photochemical efficiency when exposed to thermal or cold stress, while winter samples showed a decrease in photochemical health when subjected to thermal stress. Under cold stress, corals that were collected in the summer showed an up-regulation of actin-related protein and serine/threonine protein kinase, while corals collected during the winter did not show any cellular stress. On the other hand, under thermal stress, the most notable change was the up-regulation of phosphoenolpyruvate carboxykinase in corals that were collected during the winter season. Our observations in the differential genes expressed under temperature-derived stress suggest that *A. muricata* from Kochi may maintain physiological resilience due to the frequently encountered environmental stress, and this may play a role in the coral’s thermal tolerance.

**Electronic supplementary material:**

The online version of this article (10.1186/s13104-018-3230-z) contains supplementary material, which is available to authorized users.

## Introduction

Coral reefs are exceptionally vulnerable to climate change, as demonstrated by the increasing frequency and severity of catastrophic coral bleaching events in recent decades [1–3] including the recent devastation on the Great Barrier Reef [4]. Therefore, concern for the future survival of the coral reefs has called into question whether corals have the capacity to acclimatize to global climate change [5, 6].

Laboratory and in situ observations lend support for acclimation as an effective mechanism for increasing thermal tolerance in corals [7–11]. The survival of scattered coral colonies during mass coral bleaching events also suggest that some corals may possess inherent tolerance to environmental and thermal stress [12, 13]. Therefore, understanding the capacity for corals to survive relative environmental extremes via their cellular resistance and resilience to stress is essential.

We conducted a transcriptomic characterization of acclimation to acute thermal and cold stress in the reef-building coral *A. muricata* to determine the response of the coral to acute temperature stress below and above their general tolerance limit in summer and winter seasons. Results from this study suggest that preconditioned corals may have the capacity to adapt to the global climate change.

## Main text

### Methods

**Fig. 1:**
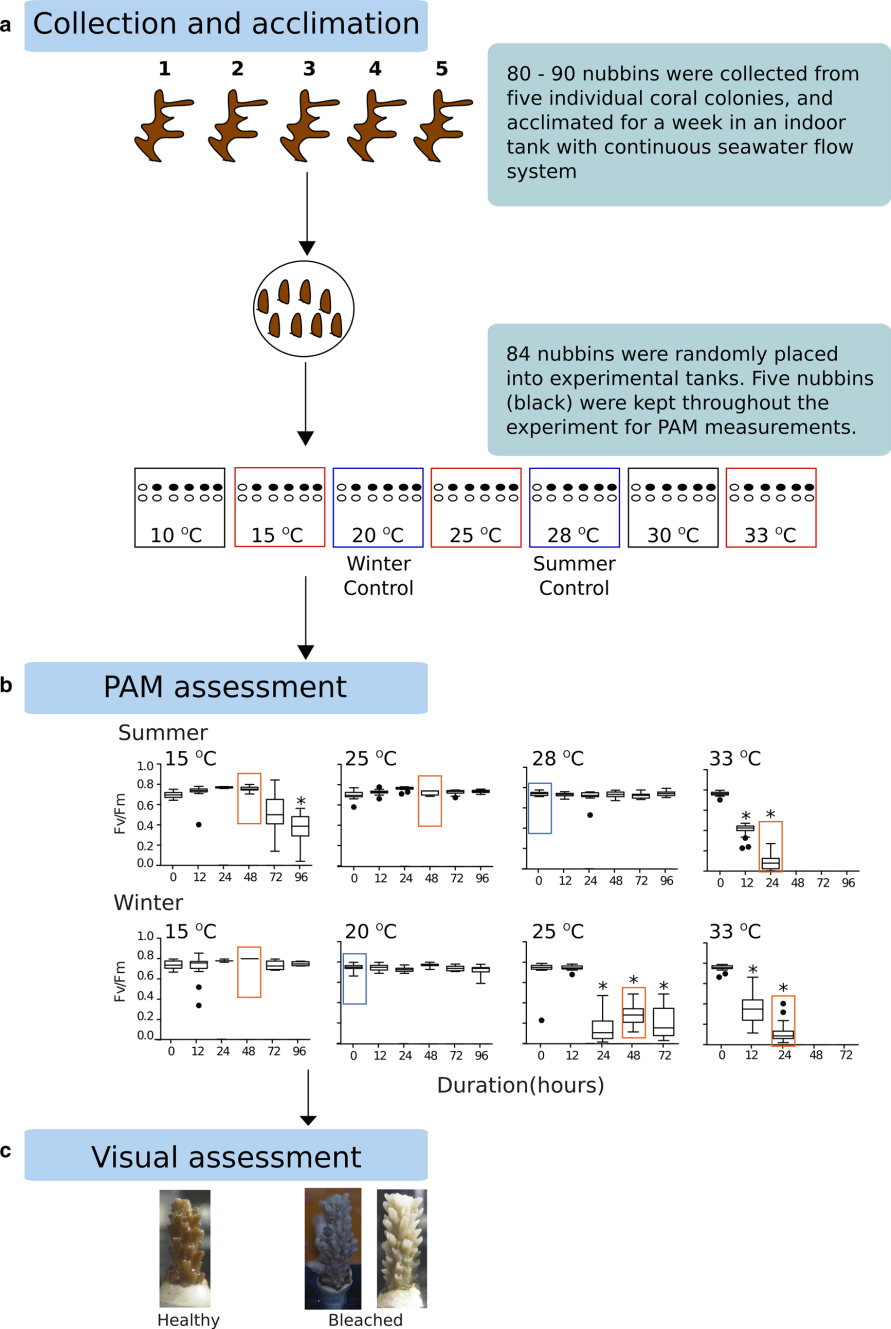
**a** Schematics of experimental design. Sample were taken from tanks that were outlined red (temperature treatment) and blue (control) (15 °C—two samples, 20 °C—one sample, 25 °C—two samples, 28 °C—one sample and 33 °C—two samples) for transcriptomic analyses. **b** Changes in the photochemical efficiency, and **c** visual assessment of *Acropora muricata* nubbins that were subjected to thermal and cold stress treatments. Samples that were enclosed in red (temperature treatment) and blue (control) rectangles were taken for transcriptomic analyses. Plot—line represents median values, boxes represent 75% percentile and whiskers represent the highest and lowest values. *Represents significant differences at *P* < 0.05 (n = 40)

We collected nubbins (~ 2–3 cm in length, n = 80–90) from five *Acropora muricata* colonies at approximately 3–4 m depth from Otsuki-Kochi, Shikoku, Japan (39°28.99′N, 141°9.00′E; Additional file 1: Figure S1) during the summer (August 2012) and winter (January 2011). After acclimation, we randomly placed the nubbins (n = 84) in seven separate acrylic experimental tanks. The coral nubbins were subjected to seven temperature treatments; (1) control treatment—28 °C (summer) and 20 °C (Winter), (2) ambient temperature—25 °C; (3) acute thermal stress—30 and 33 °C, 4. We carried out the experiment for 96 h, using a Walz^®^ Junior Pulsed Amplitude modulated (Junior PAM) fluorometer to determine the photosynthetic efficiency of *Symbiodinium* in corals during the experiments. The samples selected for transcriptome analysis were based on the visually observed response of the coral to temperature stress and through PAM measurements. Hence samples (n = 8) selected for transcriptome analysis were; 0 h control (20 °C for winter and 28 °C for summer), cold treatment—15 °C at 48 h (winter and summer), 25 °C at 48 h (winter and summer) and thermal treatment—33 °C at 24 h (summer, winter) (Fig. 1). We collected the tissue for the 33 °C thermal treatment after 24 h (instead of 48 h as per other samples) to avoid biased transcriptomic responses from the coral because of extensive tissue damage due to major bleaching of the coral sample.

Samples were fixed in TRIzol^®^ solution until RNA extraction. RNA transcriptome sequencing was carried out using Illumina HiSeq™ 2000. We assembled read pairs from the experiment using Trinity v. 2.3.0 [14], mapped the reads to the reference transcriptome using Bowtie2 v 2.1.0 [15], loaded into SAMtools v 0.1.18 [16], and quantified transcript abundance for each gene using eXpress v 1.3.0 [17].

We performed multiple comparisons with Bonferroni corrections to determine the effects of temperature on the coral physiology. In order to combat the lack of replicates for the experiment, we used the DESeq2 package [18] with the parameter—test Ward and—fitType mean, to model the biological variation of the study, defined as log_2_ of the mean difference of the genes expressed due to the treatments. We conducted a negative binomial test across all contigs to identify genes that were differently expressed at an α level of 0.01 and whose expression levels differed by onefold or more between the samples for further analysis. We also used Fisher’s exact test for functional enrichment analysis to statistically identify a particular functional category that is overrepresented or underrepresented. All COG functional groups were included so that enrichment was fully representative (Additional file 2: Methods).

### Results

The water temperature in Kochi during the winter and summer period was 16.74 ± 0.90 °C and 27.95 ± 0.63 °C respectively as recorded from HOBO data loggers installed at 5 m depth (Additional file 1: Figure S1).

There were no significant differences in *F*_*v*_*/F*_*m*_ between the control-summer (28 °C) and treatments (15, 25 °C—48 h and 33 °C) for the *A. muricata* samples that were collected during the summer. However, samples that were collected during the winter showed significant decrease in photochemical efficiency when subjected to 25°C [t(159) = 1.975, *P* < 0.001] and 33 °C [t(150) = 1.976, *P* < 0.001; Fig. 1].

**Fig. 2:**
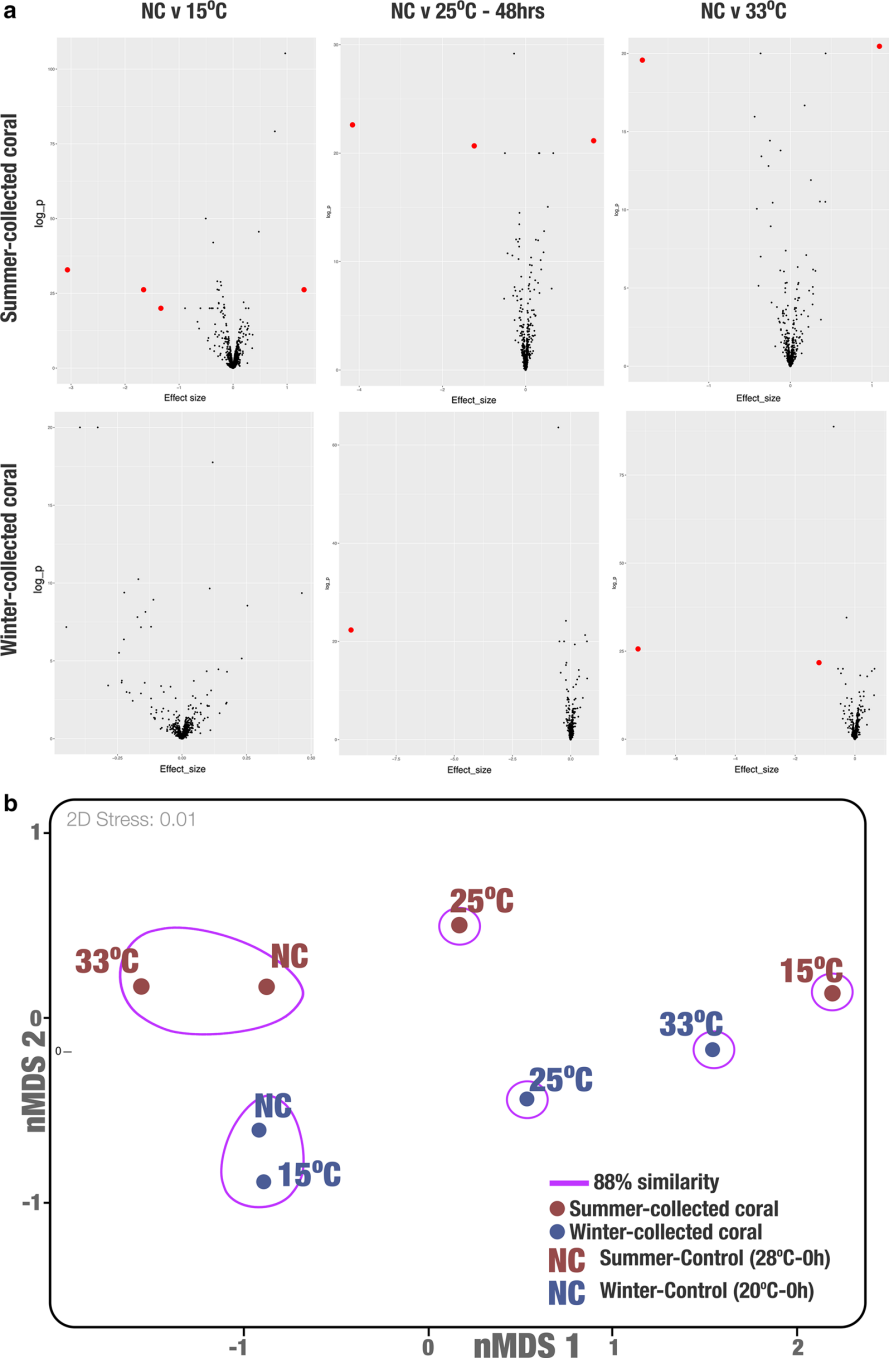
**a** Differential plots of transcriptomic analyses of summer- and winter-collected corals under different temperature treatment as compared to the control (summer—28 °C, winter 20 °C). Red dots represent significantly expressed transcripts (*P* < 0.01, effect size ≥ 1). Note that red dots are enlarged for easy visual reference. **b** Non-metric multidimensional scaling (nMDS) ordination of based on entire transcriptomic profile of coral nubbins

Coral nubbins that were collected in different seasons had different transcriptional profiles when subjected to cold and thermal stress treatments (Fig. 2). DESeq2 identified four differentially expressed genes (*P* < 0.01 and effect size ≥ 1) when the summer samples (control samples—25 °C) were subjected to cold stress (15 °C). There were three genes that were significantly different when the samples were at 25 °C after 48 h, and there were only two differentially expressed genes when samples were subjected to acute thermal stress (33 °C) (Fig. 2). We did not identify any genes that were differentially expressed significantly when winter samples (control samples—20 °C) were subjected to cold stress (15 °C). However, when subjected to thermal stress, there were genes that were significantly differentiated (25 °C—one differentially expressed gene, and 33 °C—two differentially expressed genes; Fig. 2). We used non-metric multidimensional scaling (nMDS; 2D Stress: 0.01 with Bray–Curtis similarity index) on log-normalized values of the entire transcriptomic profile, and found that the sample that was collected in the summer (0 h—28 °C) expressed similar transcripts to that when the nubbin was subjected to thermal treatment at 33 °C (88% similarity). On the other hand, nubbins collected during the winter (0 h—20 °C) were more similar to the sample that was exposed to cold stress treatment at 15 °C (Fig. 2, Additional file 3: Table S1). We observed a number of significantly enriched potential functions between the samples with an effect size of < 1 (Additional file 3: Table S1). Of 1401 identified functions, there was an enrichment of 454 and 39 functions when the summer collected nubbins were subjected to cold (15 °C) and thermal (28 °C) stress respectively. On the other hand, we observed that 38 and 186 functions were enriched when winter collected nubbins were subjected to cold (15 °C) and thermal (28 °C) stress respectively (Additional file 3: Table S1).

**Fig. 3:**
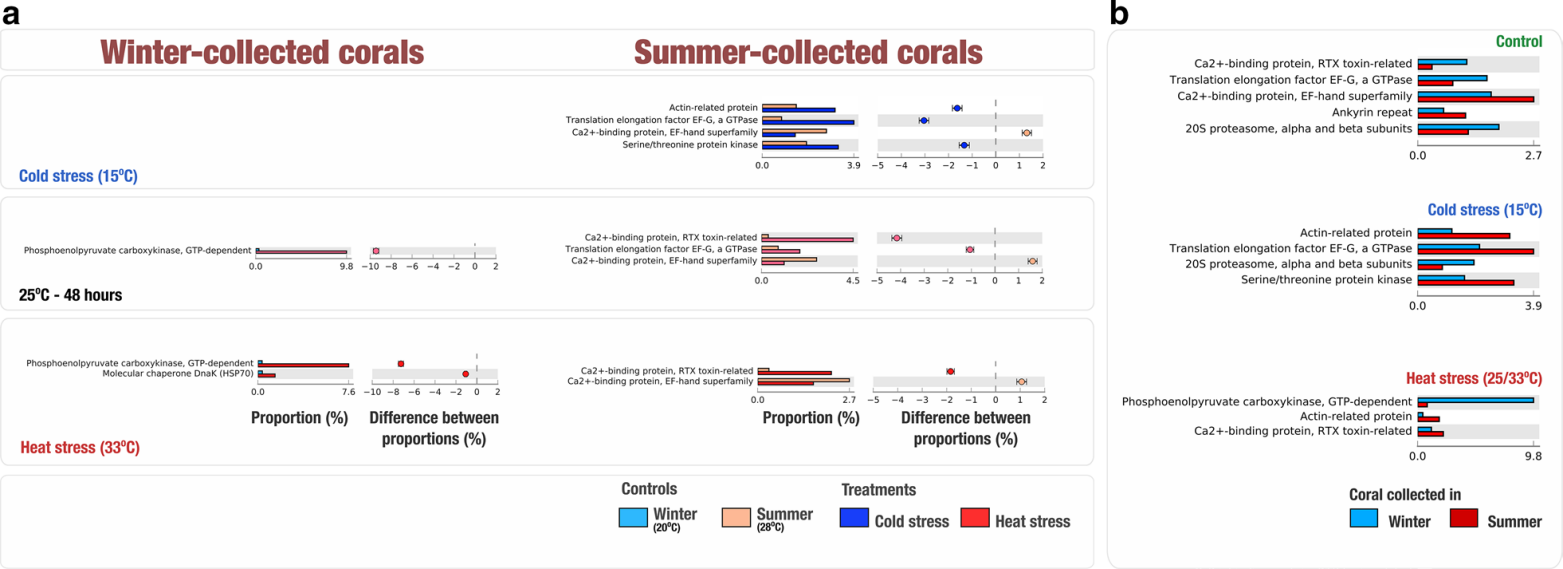
**a** Up- and Down- regulation of significant (*P* < 0.01, effect size > 1) transcripts as compared to the controlled samples (0 h) in summer- and winter-collected coral nubbins when subjected to thermal and cold stress. **b** Differential transcripts (P < 0.01, effect size > 1) in summer- and winter-collected samples when subjected to cold and thermal stress

Coral nubbins that were collected during the summer showed a significant increase (*P* < 0.01) in genes related to actin-related protein (effect size = 1.835), translation elongation factor EF-G (effect size = 3.042) and serine/threonine protein kinase (effect size = 1.336), as well as a significant drop in genes related to the biosynthesis of Ca^2+^-binding protein (effect size = 1.314) when subjected to cold stress treatment (15 °C) (Fig. 3a). In the 25 °C—48 h treatment, coral samples showed a significant increase (*P* < 0.01) in Ca^2+^-binding protein related to RTX toxin (effect size = 4.160) and translation elongation factor EF-G (effect size = 1.059) but a decrease in genes related to the biosynthesis of Ca^2+^-binding protein (effect size = 1.593). When exposed to 33 °C, nubbins showed an increase in Ca^2+^-binding protein related to RTX toxin (effect size = 1.840) and a decrease in Ca^2+^-binding protein belonging to EF-hand superfamily (effect size = 1.061) (Fig. 3a). Coral nubbins collected during the winter did not have any significant changes in expressed genes (*P* < 0.01, effect size ≥ 1) when subjected to cold stress (15 °C) treatment. However, when subjected to 25 °C, there was a significant increase (*P* < 0.01, effect size = 9.438) in genes related to phosphoenolpyruvate carboxykinase (Fig. 3a). When exposed to 33 °C, winter coral samples expressed a significant increase (*P* < 0.01) in phosphoenolpyruvate carboxykinase (effect size = 7.254) and molecular chaperone DnaK (HSP70) (effect size = 1.070) (Fig. 3a).

We further compared the transcripts expressed between samples that were collected in both seasons. Coral samples that were collected during the summer (0 h—28 °C) expressed genes related to Ca^2+^-binding protein belonging to EF-hand superfamily (*P* < 0.01, effect size = 0.992), while winter samples (0 h—20 °C) expressed genes related to Ca^2+^-binding protein that are related to RTX toxin (*P* < 0.01, effect size = 0.811) (Fig. 3b). Interestingly, when subjected to 25 °C for 48 h, nubbins that were collected in winter expressed higher relative abundance of genes related to phosphoenolpyruvate carboxykinase (effect size = 8.979) as compared to nubbins that were collected in summer when exposed to 33 °C. On the other hand, when exposed to cold stress (15 °C), nubbins collected during the summer expressed higher relative abundance of genes involved in the biosynthesis of actin-related protein (effect size = 1.926), translation elongation factor EF-G (effect size = 1.798) and serine/threonine protein kinase (effect size = 1.640) compared to winter samples, (Fig. 3b).

### Discussion

Many of the gene expression and potential functional changes we observed in this study were of small magnitude and not statistically significant. We were also unable to detect any transcriptomic changes for the winter samples subjected to cold stress at 15 °C. It is also worth noting that the number of enriched functions was higher when summer collected samples were subjected to cold as compared to thermal stress, while winter collected samples exhibited the opposite pattern. We hypothesized that this could indicate the regulation of post-transcriptional genes at a lower level of stress or represent the technical limit of this study. However, small changes in gene expression has been previously demonstrated to be of physiological relevance in the study of sexual maturation in the brains of salmon [19], and stress handling of trout [20]. As such, the small transcriptional changes observed in this study could be due to physiological fine-tuning on the part of the host.

We detected an up-regulation of phosphoenolpyruvate carboxykinase (PEPCK) only in corals that were collected in the winter, when subjected to thermal stress (25 and 33 °C), which was consistent with a decrease in the photochemical efficiency of the coral at high temperature. It was suggested that increased expression of PEPCK in bleached corals could indicate that coral hosts are making up for the loss of symbiont-derived nutritional products by converting their internal energy stores to carbohydrates [21–24]. Notably, *hsp70* transcription was also up regulated when corals that were collected in the winter were subjected to 33 °C. An up-regulation of *hsp*70 expression under elevated temperatures has also been observed in *A. millepora* larvae and embryos of *Montastraea faveolata* [25, 26]. The up-regulation of PEPCK and *hsp*70 expression in winter-collected corals but not in summer-collected samples suggests that thermal acclimatization of the coral host to high temperatures in the summer could have increased the resistance of *A. muricata* to thermal stress.

When subjected to 15 °C, corals collected during the summer showed up-regulation of actin-related protein and serine/threonine protein kinase. Over-expression of actin-related proteins suggests that changes in cytoskeletal interactions were occurring when the warmer water acclimatized coral was subjected to cold stress, which could have profound effects on the plasma membrane and transportation of lipids and proteins across the membrane [27]. The up-regulation of serine/threonine protein kinase was seen in similar studies where there was an over-expression of the gene in bleached *Acropora hyacinthus* [28] and thermally stressed *Symbiodinium* [29]. Serine/threonine protein kinases are crucial components of diverse signaling pathways and for regulation of meiosis and apoptosis. Linking the physiological deterioration of the coral and the up-regulation of these genes suggests that the coral host was responding molecularly to the cold stress to minimize damage, and re-establish cellular homeostasis [30]. The absence of physiological change and serine/threonine protein kinases expression in the winter-collected samples when subjected to cold stress indicate that *A. muricata* could possibly had acclimatized to the colder temperature and not suffered adverse effects from the treatment.

## Limitations

Although our data are only based on a limited number of samples, previous studies have suggested acclimation of corals can produce changes in gene expression responding to thermal stress and physiological functions, which enable corals to resist the impact of ocean warming [10, 31, 32]. Furthermore, collection of coral tissue for the 33 °C thermal treatment at different time makes it difficult to distinguish death-related and temperature-induced phenomena. Future projects should employ highly replicated designs to further resolve the understanding of coral acclimation.

## Additional files

**Additional file 1: Figure S1.** Location and variation of seawater temperature (SST) at the site in Kochi where the *Acropora muricata* samples were collected. The maps were drawn using the software Magic Maps Ver. 1.4.3 and Adobe Illustrator CS5 (Macintosh version) (http://magicmaps.evanmiller.org/). Permission was obtained for usage of map.

**Additional file 2: Methods.** Detailed explanation of methods in this study.

**Additional file 3: Table S1.** Assembly results of transcripts and statistical analyses of transcripts between different samples.

## Abbreviations

PAM: pulsed amplitude modulated
nMDS: non-metric multidimensional scaling
PEPCK: phosphoenolpyruvate carboxykinase
