# Supplementary figures and images for: Transcriptomic response in Acropora muricata under acute temperature stress follows preconditioned seasonal temperature fluctuations
Source: BMC Res Notes. 2018 Feb 9;11:119. doi: 10.1186/s13104-018-3230-z (PMC5807827; doi:10.1186/s13104-018-3230-z)

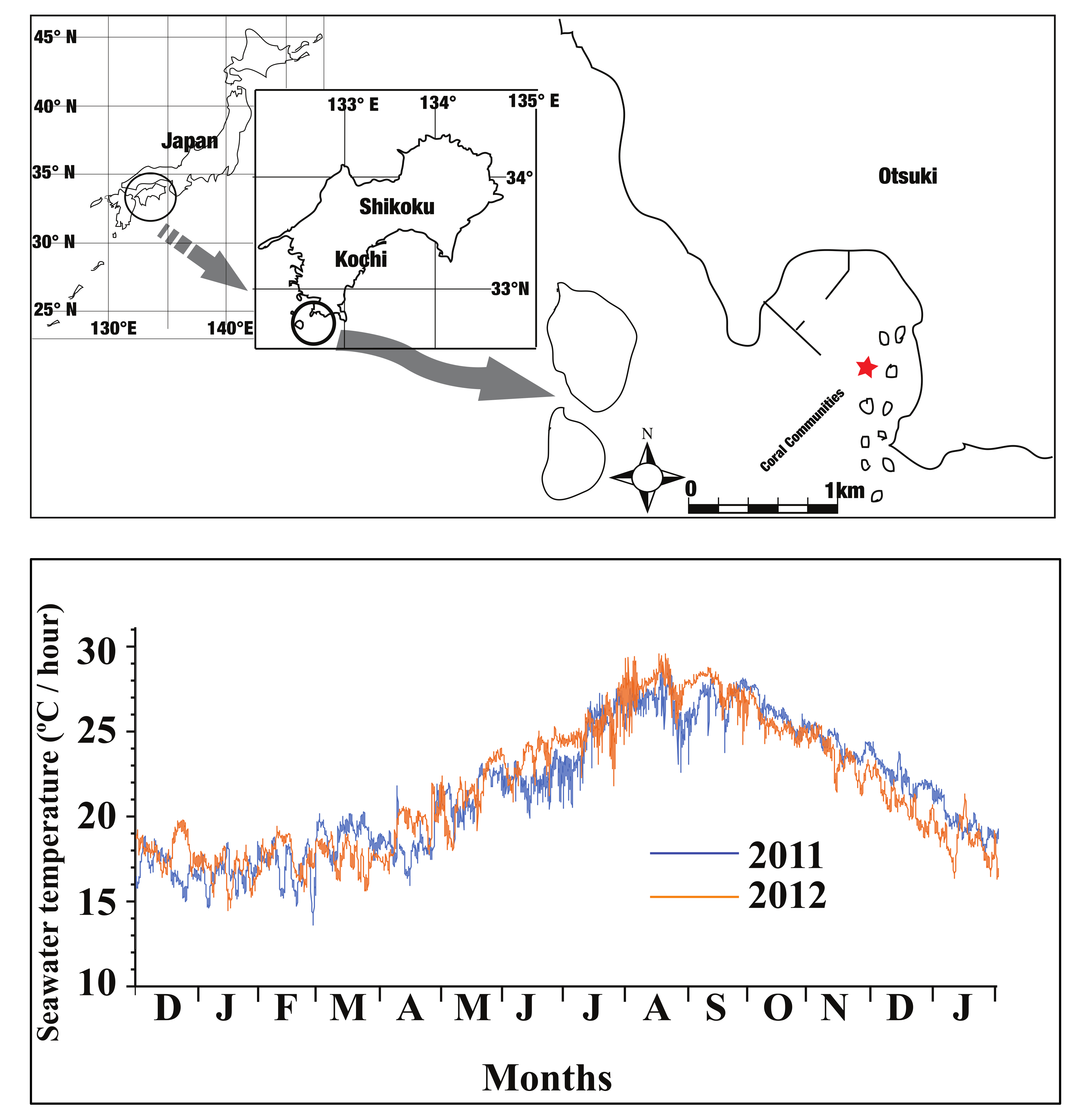

Supplement: Supplementary file 1 — Additional file 1: Figure S1. Location and variation of seawater temperature (SST) at the site in Kochi where the Acropora muricata samples were collected. The maps were drawn using the software Magic Maps Ver. 1.4.3 and Adobe Illustrator CS5 (Macintosh version) (http://magicmaps.evanmiller.org/). Permission was obtained for usage of map. [file 13104_2018_3230_MOESM1_ESM.jpg]
