# Supplementary material for: Transcriptomic response in Acropora muricata under acute temperature stress follows preconditioned seasonal temperature fluctuations
Source: BMC Res Notes. 2018 Feb 9;11:119. doi: 10.1186/s13104-018-3230-z (PMC5807827; doi:10.1186/s13104-018-3230-z)
Supplement: Supplementary file 2 — Additional file 2: Methods. Detailed explanation of methods in this study. [file 13104_2018_3230_MOESM2_ESM.docx]

**Methods**

*Study site, sample collection and experimental design*

We collected nubbins (~ 2 – 3 cm in length, n = 80 – 90) from five *Acropora muricata* colonies at approximately 3 – 4 m depth from Otsuki-Kochi, Shikoku, Japan (39°28.99’ N, 141°9.00’E; Figure S1) during the summer (August 2012) and winter (January 2011). The nubbins were then transferred to an offshore lab and acclimatized for a week in collection tanks with running seawater (0.2 µm filtered seawater flow-through and constant water temperature of 28°C for the summer and 20°C for the winter experiment). We then randomly placed the nubbins (n = 84) in seven separate acrylic experimental tanks (15 x 15 x 15 cm), which were kept in a water bath that maintained required temperature with a semi-closed flow system. The corals nubbins were subjected to seven temperature treatments; 1. Control treatment – 28ºC (summer) and 20ºC (Winter), 2. Ambient temperature – 25ºC; 3. Acute thermal stress – 30 and 33ºC, 4. Acute cold stress – 10 ºC and 15ºC. HOBO tidbit temperature-light sensor logger (HOBO corporation, USA) were placed into each of the experimental tanks and water baths to record the data at 10 min intervals throughout the experiment, with an irradiance of 100 - 120 µEm^-2^ on a 12 h light/dark cycle. We used a Walz^®^ Junior Pulsed Amplitude modulated (Junior PAM) fluorometer to determine the photosynthetic efficiency of *Symbiodinium* in corals during the experiments by measuring the *chlorophyll a* fluorescence emission by Photosystem II, using short, saturating pulses of light emitted onto photosynthetic active surfaces in relation to the fluorescence signal of continuously emitted light [1,2]. The junior PAM settings were; Measurement light intensity – 6, Frequency – 2, Saturation pulse intensity – 12, Width 0.6, System parameters Gain – 1 and ETR-F – 0.84. For each colony in every experimental tank, 5 nubbins were kept constant for PAM measurements throughout the experiment resulting in twenty-five dark-adapted yield values (F_v_/F_m_) obtained from each tank two hours after sunset. The number of PAM readings was between 25 – 5 from 0 h to 96 h due to reduction in the number of nubbins as a result of bleaching through time depending on the temperature treatment. We carried out the experiment for 96 h, and nubbin sampling (one nubbin from each tank i.e., five for each treatment for each colony) for physiological and molecular analysis was done at 0, 12, 24, 48, 72 and 96 h. Part of the nubbin was fixed in 2 ml TRIzol^®^ (Life Technologies, Grand Island, NY, USA) for later extraction of RNA. The samples selected for transcriptome analysis was based on the response of the coral to temperature stressed observed visually and through PAM measurements. Hence samples (n = 8) selected for transcriptome analysis were; 0 h control (20 ºC for winter and 28 ºC for summer), cold treatment – 15 ºC at 48 h (winter and summer), 25 ºC at 48 h (winter and summer) and thermal treatment – 33 ºC at 24 h (summer, winter) (Figure 1).

*cDNA library preparation, sequencing*

mRNAs from all eight samples were sequenced, with the hypothesis that mechanisms of the coral acclimation might differ between different seasons; meaning corals were anticipated to face more severe stress where the treatment environment was different from the local seawater temperature.

*RNA isolation and cDNA synthesis*

Samples fixed in TRIzol^®^ solution were used for RNA extraction. We used one ml from each sample for the extraction. To each sample (1 ml TRIzol^®^ solution), 200 µl of chloroform was added, vortexed for 15 secs and incubated for 3 minutes at room temperature. The solution was further centrifuge at 13,000 g for 8 minutes. We then transferred the aqueous phase in the solution to a new 2 ml Eppendorf tube, and appended with equal volume of chloroform, vortexed for 15 secs and incubated for 3 minutes. We repeated this aqueous phase and chloroform steps one more time, before adding an equal volume of 100% isopropanol and half volume of 7.5 M NH_4_OAc to the solution, incubated for 10 minutes at room temperature and centrifuged at 13,000 g for 8 minutes. The resulting RNA pellet was washed twice with chilled 75% ethanol and centrifuged at 13,000 g for 8 minutes. The RNA pellet for finally air dried and dissolved in 65 µl nuclease free ddH_2_O and stored at -80 ºC till further processing.

*Sequencing*

The RNA transcriptome sequencing was carried out by Yourgene Bioscience, Taiwan. mRNA molecules with poly-A tails were purified using poly-T oligo-attached magnetic beads from eukaryote. The cDNA was synthesized by using random Hexamer priming. The second-strand was generated to create double-strand cDNA. The cDNA was then purified by the use of Qiagen Purification Kit followed by performing end repair and A-tailing. After these procedures, the library was sequenced using Illumina HiSeqTM 2000. We then converted the quality score (Q) from the sequencing reads to error probability, and calculated the error probability (0.05) for every base. If the sum drops below zero, it is set to zero. We retained the part of the sequence between the first positive value of the running sum and the highest value of the running sum, and trimmed off everything before and after this region. In addition, we also discarded reads with length shorter than 35bp.

*Read quality control, assembly and annotation*

We used SolexaQA [3] to check the global quality of the ~100-bp transcript reads and trimmed each to its longest contiguous read segment. Only sequences with a Phred score >20 were assembled. We assembled read pairs from the experiment using Trinity v. 2.3.0 [4] using the following script ../ Trinity --seqType fa --max_memory 50G –single AM_0H_S.fa --CPU 40 --output AM_0H_S_trinity. Short contigs (<200bp) were discarded and remaining contigs were clusterd using cd-hit-est [5] (‘-c 0.95 -M 0 -T 40 -r 0;’ i.e., a 95% similarity threshold) into unique transcripts. We further used Transdecoder (from Trinity) [6] to identify and translate open reading frames (ORFs), and discarded protein sequences which was < 30 amino acids (AA). We only analyzed the longest continuous AA sequence generated from a contig, using Blastp (*e*-value cut-off of 10^-10^) from the NCBI’s nr database. KEGG was then used to gain more insight into the pathways and signal transduction interactions represented in the assembly, and functionally annotated the conceptually translated proteins. The HMM algorithm was used to search for Pfam domains in the AA sequences, and HMMER [7] was used to acquire functional modules of AA sequence domains, with a cut-off *e*-value < 10^-2^. We then used Gene onotology (GO) [8] annotation to assign GO tags to the translated proteins. GOs were assigned to the host coral contigs found to be expressed at significantly different (*P* < 0.05) levels between treatments. We mapped the reads to the reference transcriptome using Bowtie2 v 2.1.0 [9], loaded into SAMtools v 0.1.18 [10], and quantified transcript abundance for each gene using eXpress v 1.3.0 [11].

*Statistical analyses*

We performed repeated-measures ANOVA to determine the effects of temperature on the coral physiology (dark-adapted yield values, F_v_/F_m_). In order to combat the lack of replicates for the experiment, we used DESeq2 package with the parameter –test Ward and –fitType mean, to model the biological variation of the study, defined as log_2_ of the mean difference of the genes expressed due to the treatments. We conducted a negative binomial test across all contigs to identify genes that were differently expressed at an α level of 0.01 and whose expression levels differed by 1-fold or more between the samples for further analysis. We used two-sample proportion tests to determine whether the proportions of the major functions differed significantly (*P* < 0.01) between the respective treatments, and to combat the issue of generating false-positive results due to numerous statistical tests, a Bonferroni-adjusted α level was used. We also used Fisher’s exact test for functional enrichment analysis to statistically identify a particular functional category that is overrepresented or underrepresented. All COG functional groups were included so that enrichment was fully representative.

**References**

1. Schreiber U, Schliwa U, Bilger W. Continuous recording of photochemical and non-photochemical chlorophyll fluorescence quenching with a new type of modulation fluorometer. Photosynth. Res. 1986;10:51–62.

2. van Kooten O, Snel JFH. The use of chlorophyll fluorescence nomenclature in plant stress physiology. Photosynth. Res. 1990;25:147–50.

3. Cox MP, Peterson DA, Biggs PJ. SolexaQA: At-a-glance quality assessment of Illumina second-generation sequencing data. BMC Bioinformatics. 2010;11:485.

4. Grabherr MG, Haas BJ, Yassour M, Levin JZ, Thompson DA, Amit I, et al. Full-length transcriptome assembly from RNA-Seq data without a reference genome. Nat Biotech. 2011;29:644–52.

5. Huang Y, Niu B, Gao Y, Fu L, Li W. CD-HIT Suite: a web server for clustering and comparing biological sequences. Bioinformatics. 2010;26:680.

6. Haas BJ, Papanicolaou A, Yassour M, Grabherr M, Blood PD, Bowden J, et al. De novo transcript sequence reconstruction from RNA-seq using the Trinity platform for reference generation and analysis. Nat. Protoc. 2013;8:1494–512.

7. Finn RD, Clements J, Eddy SR. HMMER web server: interactive sequence similarity searching. Nucleic Acids Res. 2011;39:W29–31.

8. Ashburner M, Ball CA, Blake JA, Botstein D, Butler H, Cherry JM, et al. Gene Ontology: Tool for The Unification of Biology. Nat. Genet. 2000;25:25–9.

9. Langmead B, Salzberg SL. Fast gapped-read alignment with Bowtie 2. Nat Meth. 2012;9:357–9.

10. Li H, Handsaker B, Wysoker A, Fennell T, Ruan J, Homer N, et al. The Sequence Alignment/Map format and SAMtools. Bioinformatics. 2009;25:2078–9.

11. Roberts A, Pachter L. Streaming fragment assignment for real-time analysis of sequencing experiments. Nat Meth. 2013;10:71–3.

12. Love MI, Huber W, Anders S. Moderated estimation of fold change and dispersion for RNA-seq data with DESeq2. Genome Biol. 2014;15:550.
